# Pcdhβ deficiency affects hippocampal CA1 ensemble activity and contextual fear discrimination

**DOI:** 10.1186/s13041-020-0547-z

**Published:** 2020-01-20

**Authors:** Hirotaka Asai, Noriaki Ohkawa, Yoshito Saitoh, Khaled Ghandour, Emi Murayama, Hirofumi Nishizono, Mina Matsuo, Teruyoshi Hirayama, Ryosuke Kaneko, Shin-ichi Muramatsu, Takeshi Yagi, Kaoru Inokuchi

**Affiliations:** 1 Department of Biochemistry, Graduate School of Medicine and Pharmaceutical Sciences, University of Toyama, Toyama, 930-0194 Japan; 2 Core Research for Evolutional Science and Technology (CREST), Japan Science and Technology Agency (JST), University of Toyama, Toyama, 930-0194 Japan; 3 Precursory Research for Embryonic Science and Technology (PRESTO), JST, Saitama, 332-0012 Japan; 4 Division of Animal Experimental Laboratory, Life Science Research Center, University of Toyama, Toyama, 930-0194 Japan; 5 Department of Anatomy and Developmental Neurobiology, Tokushima University, Tokushima, 770-8501 Japan; 6 Bioresource Center, Gunma University Graduate School of Medicine, Gunma, 371-8511 Japan; 7 Division of Neurology, Department of Medicine, Jichi Medical University, Tochigi, 329-0498 Japan; 8 Center for Gene and Cell Therapy, The Institute of Medical Science, The University of Tokyo, Tokyo, 108-8639 Japan; 9 KOKORO-Biology Group, Laboratories for Integrated Biology, Graduate School of Frontier Biosciences, Osaka University, Osaka, 565-0871 Japan

**Keywords:** Hippocampus, CA1, Clustered protocadherin, Pcdhβ, Ca^2+^ imaging, Ensemble, Contextual fear memory, Discrimination

## Abstract

Clustered protocadherins (Pcdhs), a large group of adhesion molecules, are important for axonal projections and dendritic spread, but little is known about how they influence neuronal activity. The Pcdhβ cluster is strongly expressed in the hippocampus, and in vivo Ca^2+^ imaging in Pcdhβ-deficient mice revealed altered activity of neuronal ensembles but not of individual cells in this region in freely moving animals. Specifically, Pcdhβ deficiency increased the number of large-size neuronal ensembles and the proportion of cells shared between ensembles. Furthermore, Pcdhβ-deficient mice exhibited reduced repetitive neuronal population activity during exploration of a novel context and were less able to discriminate contexts in a contextual fear conditioning paradigm. These results suggest that one function of Pcdhβs is to modulate neural ensemble activity in the hippocampus to promote context discrimination.

## Introduction

Clustered protocadherins (Pcdhs) are a large group of adhesion molecules important for axonal projections and dendritic spread [1–6]. Pcdhs comprise three subgroups, Pcdhα, Pcdhβ, and Pcdhγ, expressed as 58 isoforms in mice [7]. These molecules impart two unique characteristics: each cell can express one of diverse combinations of isoforms, and the isoforms have highly specific homophilic interactions with each other [8]. Pcdhs contribute to serotonergic axonal tiling in the hippocampus, axonal projections in the olfactory bulb, dendritic self-avoidance, and dendritic spread in the retina and the cortex [1–6, 9]. However, it is not clear whether these largely morphological effects impact neuronal activity.

Pcdhαs and Pcdhγs have extracellular and intracellular domains, whereas Pcdhβs have only extracellular domains [10]. Pcdhβs are strongly expressed in the olfactory bulb [11], influencing axonal targeting during embryonic stage [12], as well as in the hippocampus, a brain region in which synchronous, or ensemble, activity of neurons enables learning and memory [13, 14]. However, whether Pcdhβs affect ensemble activity in the hippocampus is not known. Of note, synchronous activity is regulated by inhibitory GABAergic neurons [15, 16], and the loss of Pcdhβs was shown to affect the survival of spinal inhibitory neurons during embryonic stage [12].

Here, we investigated neural activity in the hippocampi of Pcdhβ-deficient mice. In vivo Ca^2+^ imaging revealed that a loss of Pcdhβ induced distinctive neural ensemble activity in the CA1 region during context exploration. This resulted in impaired contextual discrimination during behavioral experiments involving fear conditioning.

## Results

### Pcdhβ deficiency reduces population activity in hippocampal CA1

**Fig. 1:**
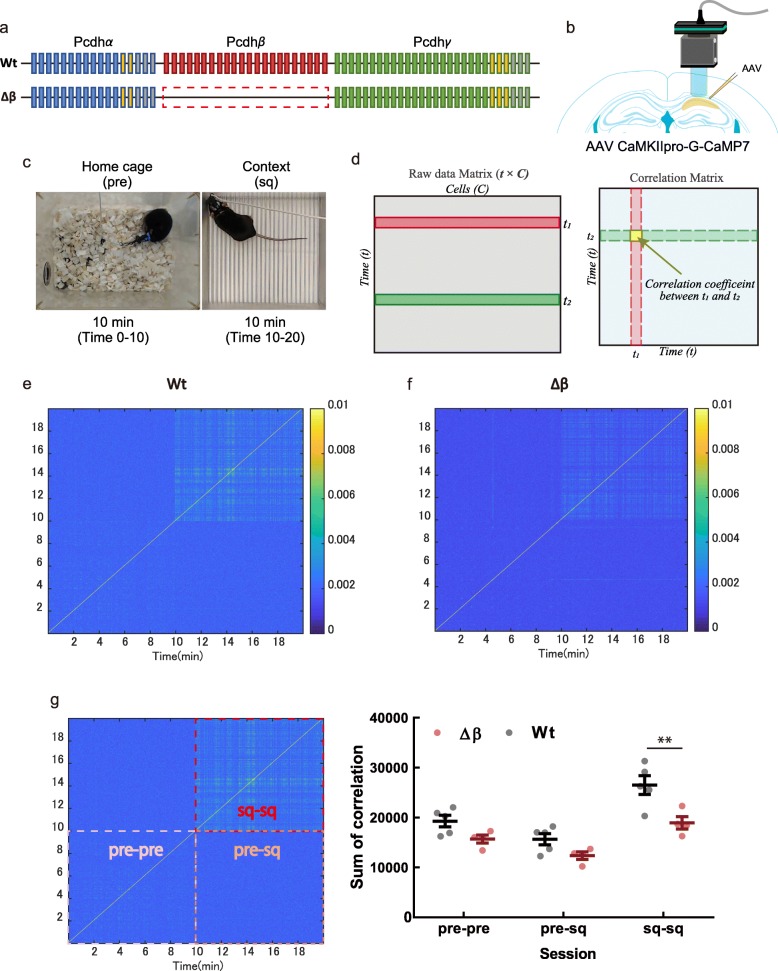
Pcdhβ deficiency reduces repetitive neuronal activity in the hippocampus. **a** Pcdhβ deletion. **b** Induction of CaMKII-G-CaMP7 by AAV and implantation of GRIN lens over the right hippocampal CA1 region. **c** Ca^2+^ imaging in home cage (pre) and square context (sq). **d** Schematic of the correlation matrix analysis. Representative images of correlation matrices for activation in Wt (**e**) and Δβ (**f**) mice. **g** Summation of correlation coefficients between sessions (*n* = 5 Wt mice, 4 Δβ mice). Data are means ± standard errors of the means (SEMs). **P < 0.01 (adjusted *P*-value from Bonferroni’s multiple-comparison test)

To investigate the effect of Pcdhβ deletion on hippocampal neuronal activity, we performed Ca^2+^ imaging experiments in freely moving wild-type (Wt) and Pcdhβ-deficient (Δβ) mice (Fig. 1a). For this, neurons in CA1 were infected with adeno-associated viruses (AAVs) encoding calcium indicator G-CaMP7 under the control of the CaMKII promoter for expression by excitatory neurons, and the mice were fitted with a head-mount miniature fluorescence microscope [14, 17, 18] (Fig. 1b). Ca^2+^ imaging was performed while mice were in their home cage (pre) and during contextual exploration (sq) sessions [14, 19] (Fig. 1c). Acquired images were then processed and analyzed as described previously [14] (see Additional file 1: Figure S1). Correlation matrix analysis (Fig. 1d) showed that repetitive neuronal activity in Δβ mice was reduced during sq. sessions compared with that in Wt mice at different time points (Fig. 1e–f). The sum of the correlations during contextual exploration (sq-sq) was significantly lower in Δβ mice than in Wt mice (Fig. 1g; adjusted *P*-value (*P*_adj_) = 0.0013 by Bonferroni’s multiple-comparison test). However, no significant difference in the repetitive correlation activity was observed either in home cage (pre-pre) sessions or between home cage and context exposure (pre-sq) sessions in Δβ or Wt mice (Fig. 1g; *P*_adj_ = 0.1838 for pre-pre, *P*_adj_ = 0.2583 for pre-sq by Bonferroni’s multiple-comparison test). The difference observed during contextual exploration was not due to their difference of behavioral activity in both genotypes because their behavioral activity was equivalent in open field task (Additional file 2: Figure S2). Moreover, there were no significant differences between Wt and Δβ mice at the individual cell level regarding the number of observed Ca^2+^ events or ratios of sq./pre Ca^2+^ events (Additional file 3: Figure S3). These results suggest that Pcdhβ deficiency reduces repetitive synchronous activity in the CA1 at the population level but not at the cellular level during contextual exploration.

### Pcdhβ deficiency modulates ensemble activity in CA1

**Fig. 2:**
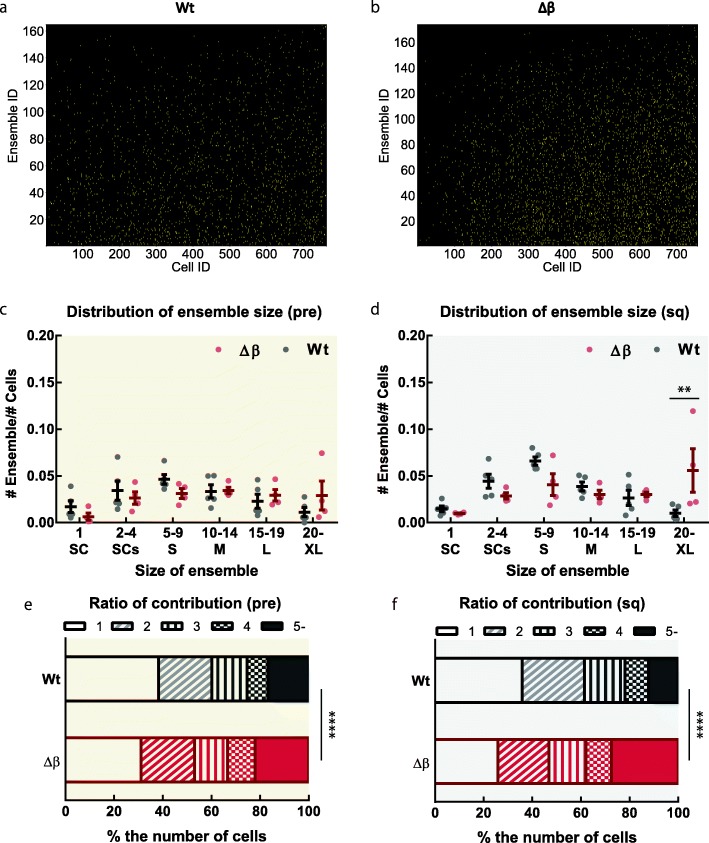
Pcdhβ deficiency affects ensemble size and the proportion of neurons shared between ensembles. Representative images of binarized basis matrices from Wt (**a**) and Δβ (**b**) mice. Yellow dots represent the cells contributing to the ensemble. **c–d** Normalized number of ensembles according to ensemble size. Statistical values from Bonferroni’s multiple-comparison test are provided in Additional file 6: Table S1 (*n* = 5 Wt mice, 4 Δβ mice). Data are means ± SEMs. **P < 0.01 (adjusted *P*-value from Bonferroni’s multiple-comparison test). Percentages of neuronal cells contributing to one ensemble (open bars) and multiple ensembles (pattern and solid bars) in Wt and Δβ mice during pre (**e**) and sq (**f**) sessions. ****P < 0.0001 (chi-square test)

To further study the effect of Pcdhβ deletion on population activity, we used non-negative matrix factorization (NMF) to analyze ensembles of collectively firing neurons [14] (Additional file 4: Figure S4a–b). The normalized number of total ensembles extracted by NMF was similar between Wt and Δβ mice in both pre and sq sessions (Additional file 4: Figure S4c–d). The number of ensembles comprising 20 or more cells, defined here as large ensembles, was larger in Δβ mice than in Wt mice (Fig. 2a–d; Additional file 5: Figure S5). However, these large ensembles were seldomly activated (Additional file 5: Figure S5). Although the number of activated cells were similar between the genotypes (Additional file 1: Figure S1c), larger portion of cells was shared across the ensembles in Δβ mice than in Wt mice in both sessions (Fig. 2e–f). These results demonstrate that Pcdhβs modulate ensemble size and composition in hippocampal CA1.

### Pcdhβ deficiency impairs context discrimination during fear conditioning

**Fig. 3:**
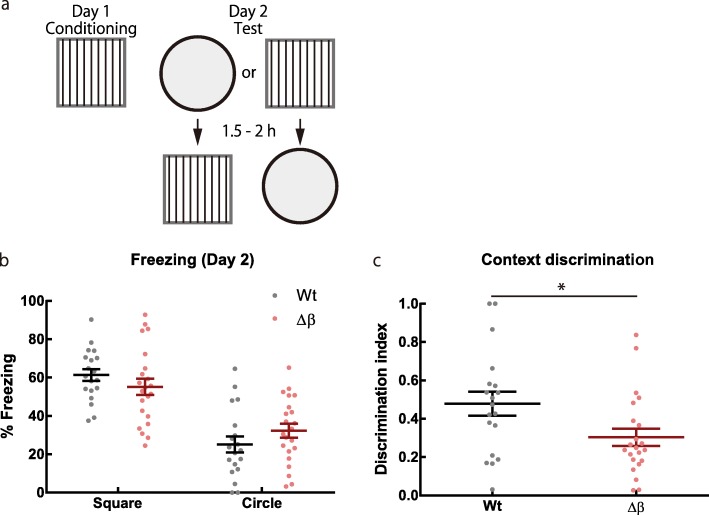
Pcdhβ deficiency impairs context discrimination. **a** Schematic image of contextual fear conditioning task. **b** Freezing levels in test sessions. Square: *F*_(21, 18)_ = 2.140, *P* = 0.1080; *t*_39_ = 1.141, *P* = 0. 2607; circle: *F*_(18, 21)_ = 1.153, *P* = 0.7479; *t*_39_ = 1.301, *P* = 0.2008; unpaired *t*-test. **c** Discrimination indices of both contexts. *F*_(18, 21)_ = 1.645, *P* = 0.2737; *t*_39_ = 2.310, *P* = 0.0263; unpaired *t*-test. (*n* = 19 Wt mice, 22 Δβ mice). Data are means ± SEMs

As Pcdhβ deficiency altered neuronal activity in the hippocampus, we investigated the effect on hippocampus-dependent memory function with contextual fear conditioning task [20] (Fig. 3a). Pcdhβ-deletion and Wt mice showed comparable levels of freezing in the conditioned context (Fig. 3b; square: *F*_(21, 18)_ = 2.140, *P* = 0.1080; *t*_39_ = 1.141, *P* = 0. 2607; unpaired *t*-test). However, the discrimination efficiency between the conditioned context (square) and a novel context (circle) was significantly impaired in Δβ mice compared with that in Wt mice (Fig. 3c; *F*_(18, 21)_ = 1.645, *P* = 0.2737; *t*_39_ = 2.310, *P* = 0.0263; unpaired *t*-test). These results suggest that Pcdhβs play a key role in the specificity of memories and in discriminating between events, which is consistent with the formation of ensembles with more shared cells in Δβ mice than in Wt mice.

## Discussion

In this study, we showed that Pcdhβs contribute to the organization of neuronal ensembles in the hippocampus of freely moving mice. These findings extend what was previously shown regarding the role of Pcdhs in regulating neuronal morphology [1–5]. Interestingly, we found that Pcdhβ regulates neuronal activity at the population level but not at the cellular level. Specifically, Pcdhβ deficiency resulted in larger ensembles in CA1 and larger proportion of neurons shared between ensembles, thereby reducing their ensemble diversity. This supports the hypothesis that Pcdhs are important for the formation of diverse assembly based on the multitude of possible combinations of isoforms, which have highly specific homophilic interactions [21]. Furthermore, the large ensembles in Δβ mice were rarely activated (Additional file 5: Figure S5). These results are consistent with those from an in vitro study showing that complete deletion of Pcdhs (including α, β, and γ isoforms) induces large-amplitude low-frequency synchronous Ca^2+^ activity [22]. Thus, Pcdhβs are important for the diversity of neuronal activity in the hippocampus.

The altered ensemble activity in the hippocampi of Δβ mice resulted in an impaired ability to discriminate contexts in a fear conditioning memory task without affecting their ability to learn the association. This is consistent with previous reports showing that spatial memory was not impaired in Pcdh-mutant mice [3, 23], although Pcdhα was important for high load memory tasks [24]. The impairment of context discrimination exhibited by the Δβ mice may be attributed to the increased sharing of cells between ensembles. As previously reported, a set of neurons or ensemble has specific information [25, 26]. These can support our interpretation that low independence of neuronal composition between ensembles in Δβ mice will lead the inability to process distinguish information about two contexts. Nevertheless, it still remains an open question how neurons are selected in allocating information. Collectively, the evidence suggests that Pcdhs play an important role in higher brain function such as context discrimination.

In summary, the data presented here demonstrate that Pcdhβs tune ensemble activities in the CA1 area of the hippocampus. Our results suggest that this ensemble activity contributes to contextual discrimination. The findings that Pcdhs configure functional ensembles helps to elucidate the mechanisms underlying the processing of information in the mammalian brain.

## Methods

### Animals

The generation of Δβ mice was described previously [12]. Briefly, in vitro fertilization and embryo transfer techniques were performed using heterozygous male and female mice [19]. Genotypes were determined from tail DNA samples by PCR using primers TTGTGAGTGCTCCATAGCCTC, GCTCCTGATTGAATTTGCC, and TGATGTGGGTCTGGTTTCC with conditions of 95 °C for 3 min, 30 cycles of 95 °C for 15 s, 60 °C for 15 s, 72 °C for 45 s, and 72 °C for 2 min. The resulting PCR products were 519 bp for Wt and 626 bp for Δβ mice.

The mice were housed under a 12 h light/dark cycle (lights on, 7:00 am) at 24 °C ± 3 °C and 55% ± 5% humidity with ad libitum access to food and water. All animal experiments were conducted in accordance with the guidelines of the National Institutes of Health (NIH) and were approved by the Animal Care and Use Committee of the University of Toyama.

### AAV constructs

The pAAV-CaMKII-G-CaMP7 plasmid was constructed by replacing C1V1-mCherry from pAAV-CaMKII-C1V1-mCherry plasmid (donated by Dr. Karl Deisseroth) with the G-CaMP7 sequence from pN1-G-CaMP7 plasmid [27, 28] using the BamHI and EcoRI sites of pAAV-CaMKII-C1V1-mCherry, and the BglII and NotI sites of pN1-G-CaMP7. The resulting pAAV-CaMKII-G-CaMP7 plasmid was prepared using an EndoFree Plasmid Maxi kit (Qiagen). The recombinant AAV vectors were produced as described previously [29, 30] to obtain a viral titer of 9.4 × 10^12^ vg/mL. The virus was diluted 40-fold in phosphate-buffered saline for injections into mice.

### Stereotactic surgery

Male Δβ mice and Wt littermates (at least 12 weeks old) were anesthetized with pentobarbital (64.8 mg/kg of body weight, intraperitoneally (i.p.)) or a combination anesthetic (0.75 mg/kg, i.p., medetomidine (Domitor; Nippon Zenyaku Kogyo Co., Ltd., Japan), 4.0 mg/kg, i.p., midazolam (Fuji Pharma Co., Ltd., Japan), and 5.0 mg/kg, i.p., butorphanol (Vetorphale, Meiji Seika Pharma Co., Ltd., Japan) [31]) and were then placed in a stereotactic apparatus (Narishige, Japan). AAV (0.3 μL) was injected into the right CA1 (2.0 mm posterior and 1.5 mm lateral to bregma, 1.45 mm below the dura) at a rate of 0.5 μL/min using a mineral oil-filled glass micropipette connected to an IMS-20 (Narishige). The glass micropipette was kept at the target site for an additional 3 min before being withdrawn. Animals anesthetized with combination anesthetic were given an injection of atipamezole (1.5 mg/kg, intramuscularly (Antisedan; Nippon Zenyaku Kogyo Co., Japan)), an antagonist of medetomidine, to promote recovery from the anesthesia.

At least 2 weeks after AAV injection, a gradient index (GRIN) lens was implanted in each mouse as described previously [14, 17, 18, 32]. Mice were anesthetized as described above and placed in a stereotactic apparatus. A craniotomy (2.0 mm diameter) was performed centered over the injection site, and the neocortex and corpus callosum above the alveus overlying the dorsal hippocampal CA1 were aspirated under constant irrigation with saline using a 26-gauge flat-tip needle. Saline was applied to control the bleeding, and the GRIN lens (1 mm diameter, 4 mm length; Inscopix, Inc., USA) was placed on the alveus and depressed an additional 10–30 μm using handmade forceps attached to a manipulator (Narishige). Bone wax melted by a low-temperature cautery was applied to seal any gaps between the skull edge and the GRIN lens, and the lens was cemented in place. Five to seven anchor screws were placed in the skull for reinforcement. After the surgery, Ringer’s solution (0.7 mL/mouse, i.p.; Otsuka, Japan) was injected, and atipamezole was administered as described above. Mice were maintained in individual cages after the surgery.

At least 3 weeks after GRIN implantation, surgery to set a baseplate (Inscopix, Inc.) was performed as described previously [14, 17]. Briefly, anesthetized mice were placed in a stereotactic apparatus, and a baseplate attached to a miniature microscope (nVista HD v2; Inscopix, Inc.) set at the middle of its focal range was arranged using Gripper (Inscopix, Inc.); the optimal location was determined according to blood vessels as cues. The baseplate was cemented to the cement over the skull at 5–20 μm above the optimal location. Atipamezole was administered to mice receiving the combination anesthetic, and the baseplate cover (Inscopix, Inc.) was placed over the GRIN lens until Ca^2+^ imaging was performed.

### Ca^2+^ imaging

Six days or more after setting the baseplate, mice were habituated to the experimental room for at least 1 day. Mice were lightly anesthetized with isoflurane to mount the miniature microscope. When the mice became quiet (resting state) at least 30 min after their recovery from the anesthesia, Ca^2+^ imaging was performed while the mice were in their home cage (habituation session). The imaging data were acquired for 10 min, and the microscope was detached after an additional 10 min. On the following day, Ca^2+^ imaging was similarly performed in the home cage (pre session) followed by Ca^2+^ imaging for 10 min in a novel square context (Muromachi Kikai, Co., Ltd., Japan; sq. session). The mice were then returned to their home cage (see Fig. 1b).

The square context had a Plexiglass front, gray sides, and back walls (width × depth × height: 175 mm × 165 mm × 400 mm), and the chamber floors comprised 26 stainless steel rods (2 mm diameter) placed 5 mm apart.

Calcium imaging was performed during the light cycle. Imaging data were acquired using nVista acquisition software (Inscopix, Inc.) at 1440 × 1280 pixels, at 20 frames/s, maximum gain and at optimal LED power (based on the histogram, according to the nVista user guide).

### Data acquisition, preprocessing, and cell identification

Calcium transients recorded by nVista acquisition software were processed with Mosaic software (Inscopix, Inc.) as described previously [14, 33]. First, each session movie was spatially downsampled and smoothed by a factor of 2. Motion correction was performed (correction type, translation only; spatial mean (*r* = 20 pixels) subtracted, and spatial mean applied (*r* = 5 pixels)) using blood vessels as a landmark to maintain the same field of view and to correct for motion artefacts across sessions using an identical reference frame. The movie was processed with Fiji software (NIH) to remove noise. Each session movie was low-pass filtered (*r* = 20 pixels).

Each session movie was cropped at the same coordinates, and the fluorescence intensity change (ΔF/F) was calculated by Mosaic as follows: ΔF/F = (F – F_m_)/F_m_, where F is the fluorescence from an individual frame of the movie and F_m_ is mean fluorescence for the entire movie for that session. These resultant ΔF/F movies were concatenated. Finally, cell identification was conducted with HOTARU, an automatic sorting system, as previously reported [14, 34, 35]. The HOTARU output data matrix ($\hat{D}$; time × neuron) represents Ca^2+^ activity in each time frame for every cell.

### Data analysis

#### Correlation matrix

To investigate repetitive synchronous activity, temporal correlation was calculated as described previously [14, 36]. Briefly, the Ca^2+^ intensity matrix ($\hat{D}$) was binned every four frames (200 ms), and then the correlation was calculated using five binned frames (1 s) as a sliding time window. For comparison between genotypes, the correlation matrix was summed every session (pre-pre, pre-sq, and sq-sq), as shown in Fig. 1g.

#### NMF

To extract ensemble activity, NMF was applied to the data matrix every session as described previously [14, 37, 38]. Briefly, $\hat{D}$ was binned every four frames (200 ms), and then NMF was adapted. As a result, $\hat{D}$ was optimally factorized into basis matrix ($\hat{B}$; neural ensemble matrix, ensemble × neuron) and the corresponding occurrence matrix ($\hat{C}$; occurrence matrix, time × ensemble), $\hat{D}\approx \hat{B}\hat{C}$. Here, $\hat{B}$ represents neuronal ensembles activated synchronously, and $\hat{C}$ represents the temporal activation intensity of the corresponding ensembles. To determine the optimal number of ensembles, the Akaike information criterion with second-order correction was used. To find the optimal factorization, the ensemble (basis) matrix and intensity (occurrence) matrix minimizing the cost function, defined by *E* ≡ ∑_*ij*_(*D*_*ij*_ − ∑_*k*_*B*_*ik*_*C*_*kj*_)^2^, were chosen to be the best factorization when random initial entries from matrices $\hat{B}$ and $\hat{C}$ were used for 1000 attempts at minimization.

For quantification, the number of ensembles were normalized by dividing the number of cells in each mouse to minimize the effect of the number of cells on the number of ensembles. For $\hat{B}$ and $\hat{C}$, contributing neurons and the active time of each ensemble were judged on the basis of the z-score in each ensemble (≥2 standard deviations (SDs) for contribution, ≥3 SDs for active time).

For quantification of activity at the cellular level, Ca^2+^ events were judged as activity ≥3 SDs for the entire session.

### Contextual fear conditioning

Male mice 12–19 weeks old were individually housed in a room adjacent to the testing room at least 7 days before behavioral experiments. Behavioral experiments were conducted during the light cycle by investigators blinded to the genotypes of the mice. Both genotypes of mice were run in parallel. For conditioning (day 1), mice were placed in a square context (context A) and were allowed to explore freely for 148 s. Then, three foot shocks (0.4 mA, 2 s duration, 1 min apart) were applied to the chamber floor grid. One minute after the last foot shock, the mice were returned to the home cages. In the test session (day 2), mice were placed in context A or context B (circle context) for 3 min; 1.5–2 h later they were placed in the other context for 3 min. A video tracking system (Muromachi Kikai Co., Ltd.) was used to measure the freezing response, in which the motion threshold was < 10 for at least 1.5 s. Discrimination indexes were calculated as follows [39]: (freezing in context A – freezing in context B)/(freezing in context A + freezing in context B).

The behavioral equipment was described previously ([14, 19, 20]). Context A was the square context described for Ca^2+^ imaging. Context B was a black cylindrical chamber (185 mm diameter, 220 mm height) with a white acrylic floor. Overhead room lights lit both contexts, and background noise was provided by a fan inside the room. The equipment was cleaned with 80% ethanol before each exposure. A white container was used to transfer mice between the room they were housed in and the experimental room for exposure to context B.

### Open field test

Male mice 12–19 weeks old were individually housed in a room adjacent to the testing room at least 7 days before behavioral experiments. Behavioral experiments were conducted during the light cycle by investigators blinded to the genotypes of the mice. Both genotypes of mice were run in parallel.

The mice were transported from the maintenance room to the experimental room and left undisturbed for at least 30 min. They were placed in open field box for 30 min. A tracking software, DuoMouse (National Institute of Genetics) [40], was used to measure their center of mass and pixel change between frames [41]. The open field box (width × depth × height: 500 mm × 500 mm × 300 mm) was with a white acrylic floor and a side wall and side walls with different pattern. Background noise was provided by a fan inside the room. The equipment was cleaned with 80% ethanol before each exposure.

### Statistical analysis

Statistical analyses were performed in GraphPad Prism 6 (GraphPad Software) and MATLAB (MathWorks). Data analyses were performed with Student’s *t*-tests, Bonferroni’s multiple-comparison tests, chi-square tests, and Kolmogorov–Smirnov tests, as appropriate. A *P*-value < 0.05 was considered significant.

## Supplementary information

**Additional file 1: Figure S1.** Pcdhβ deficiency does not affect the number of cells activated during recording (related to Figs. 1 and 2). (a) Flowchart of Ca^2+^ imaging data analysis. (b) Representative image and traces of identified cells. (c) Number of cells observed in the entire session. n.s.: not significant (unpaired *t*-test). *F*_(3, 4)_ = 1.087, *P* = 0.9005; *t*_7_ = 2.202, *P* = 0.0635 (*n* = 5 Wt mice, 4 Δβ mice). Data are means ± SEMs.

**Additional file 2: Figure S2.** Behavioral activity was equivalent in open field task. (a) Open field box. (b) Traces of center of mass. Travel distance (c) and motility (d). Panel c: *F*_(25, 27)_ = 3.434, *P* = 0.0023; *t*_37.73_ = 0.1515, *P* = 0.880; panel d: *F*_(25, 27)_ = 4.253, *P* = 0.0004; *t*_35.54_ = 0.2776, *P* = 0.783; unpaired *t*-test with Welch’s correction; *n* = 26 Wt mice, 28 Δβ mice. Data are means ± SEMs.

**Additional file 3: Figure S3.** Pcdhβ deficiency does not affect neural activity at the cellular level (related to Figs. 1 and 2, and additional file 1). (a–b) Representative images of raster plots of all recorded cells from Wt (a) and Δβ (b) mice. (c) Cumulative curve of proportion of cells against number of active events during entire recording session (pre 10 min + sq 10 min). Proportions of cells according to the number of Ca^2+^ events during pre (d) and sq (e) sessions. Statistical values from Bonferroni’s multiple-comparison test are provided in Additional file 6. (f) The ratios of the number of Ca^2+^ events during the sq session to that in the pre session. Statistical values from Bonferroni’s multiple-comparison test are provided in Additional file 6 (*n* = 5 Wt mice, 4 Δβ mice). Data are means ± SEMs. No significant differences were observed (Kolmogorov–Smirnov test for panel c, Bonferroni’s multiple-comparison tests for panels d–f).

**Additional file 4: Figure S4.** Pcdhβ deficiency does not affect the number of ensembles activated in the home cage and during novel context exploration (related to Fig. 2). (a) Schematic diagram of the non-negative matrix factorization (NMF) analysis. (b) Representative image of an ensemble extracted with NMF. Red arrows (left) indicate the cells contributing to this ensemble. Red dots (right) indicate the times at which the ensemble was activated. (c–d) Normalized number of ensembles extracted in each session (panel c: *F*_(4, 3)_ = 1.907, *P* = 0.6229; *t*_7_ = 1.648, *P* = 0.1434; panel d: *F*_(4, 3)_ = 1.086, *P* = 0.9856; *t*_7_ = 0.2852, *P* = 0.7837; unpaired *t*-test; *n* = 5 Wt mice, 4 Δβ mice). Data are means ± SEMs.

**Additional file 5: Figure S5.** Large ensembles are infrequently activated. Number of ensemble events during pre and sq sessions in extra-large ensembles (XL; 20 or more cells; a–b), large ensembles (L; 15–19 cells; c–d), medium ensembles (M; 10–14 cells; e–f), and small ensembles (S; 5–9 cells; g–h). Number of ensemble events during pre and sq. sessions in ensembles composed of several cells (SCs; 2–4 cells; i–j) and of single cells (SC; k–l). Statistical values from Bonferroni’s multiple-comparison tests are provided in Additional file 6 (*n* = 5 Wt mice, 4 Δβ mice). Data are means ± SEMs. *P < 0.05 (adjusted *P*-value from Bonferroni’s multiple-comparison test).

**Additional file 6 Table S1.** Tables of statistical values. *P*-values by statistical analysis of all figures and additional files.

## Data Availability

The datasets used and/or analyzed during the current study are available from the corresponding author on reasonable request.

## Abbreviations

AAV: Adeno-associated virus
NMF: Non-negative matrix factorization
Pcdh: Clustered protocadherin
SD: Standard deviation
SEM: Standard error of the mean
Wt: Wild type
Δβ: Pcdhβ deficient
