# Supplementary figures and images for: Pcdhβ deficiency affects hippocampal CA1 ensemble activity and contextual fear discrimination
Source: Mol Brain. 2020 Jan 20;13:7. doi: 10.1186/s13041-020-0547-z (PMC6971911; doi:10.1186/s13041-020-0547-z)

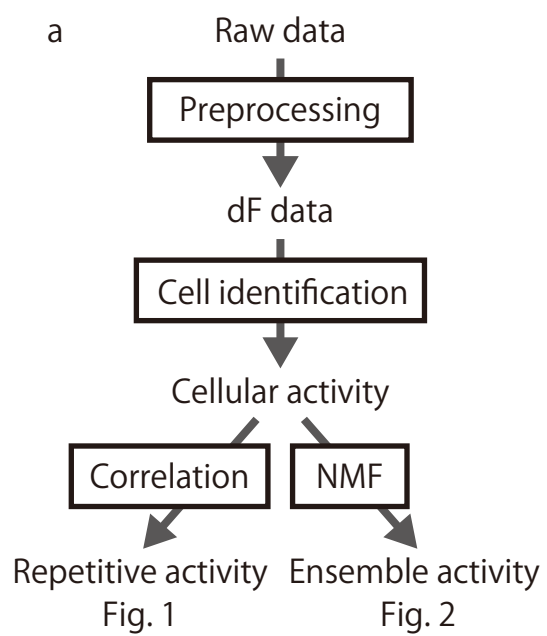

b Cell identification

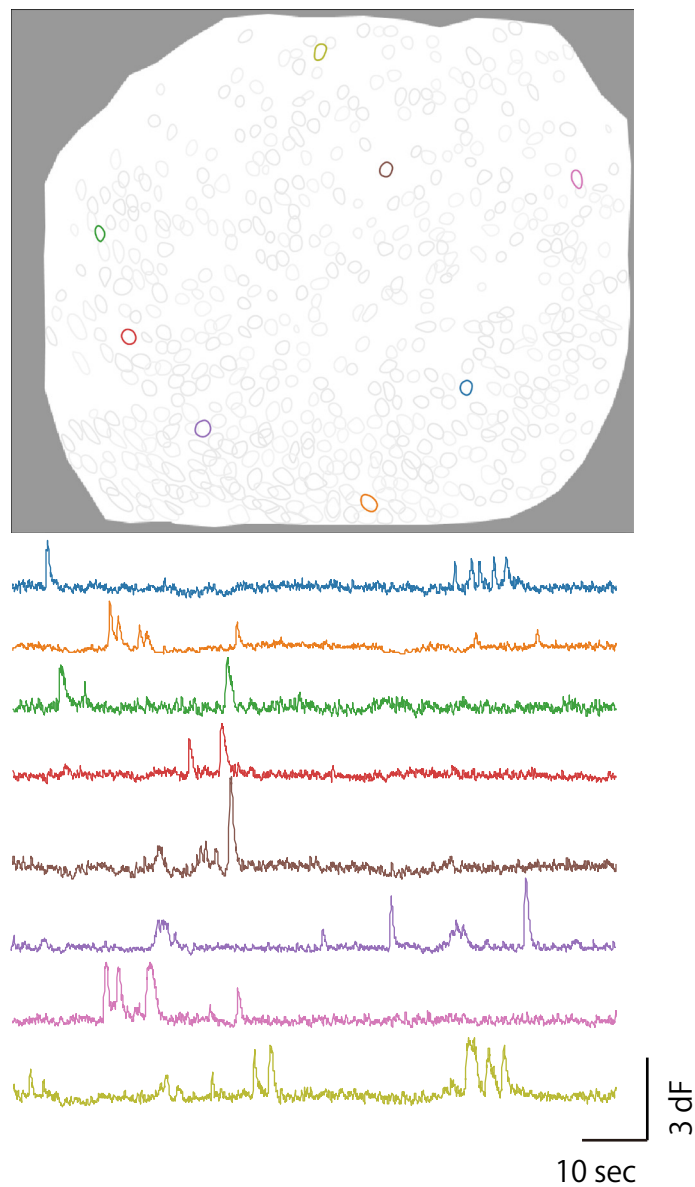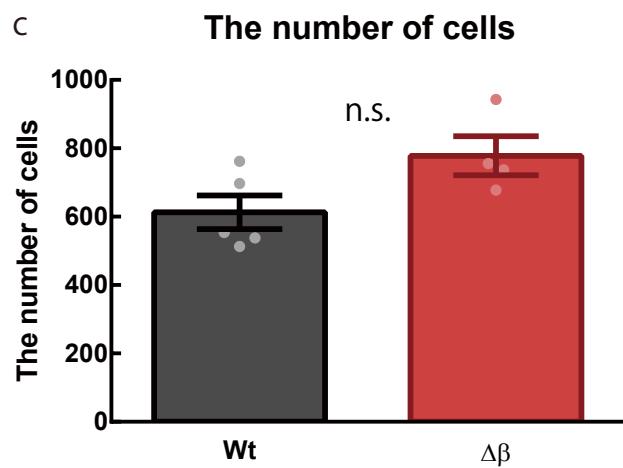

Additional file 1.

Supplement: Supplementary file 1 — Additional file 1: Figure S1. Pcdhβ deficiency does not affect the number of cells activated during recording (related to Figs. 1 and 2). (a) Flowchart of Ca2+ imaging data analysis. (b) Representative image and traces of identified cells. (c) Number of cells observed in the entire session. n.s.: not significant (unpaired t-test). F(3, 4) = 1.087, P = 0.9005; t7 = 2.202, P = 0.0635 (n = 5 Wt mice, 4 Δβ mice). Data are means ± SEMs. [file 13041_2020_547_MOESM1_ESM.pdf]

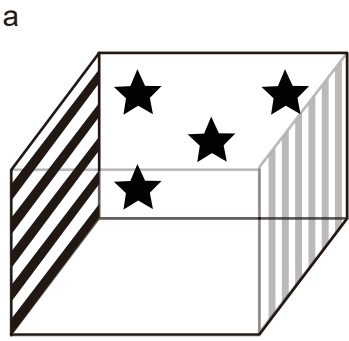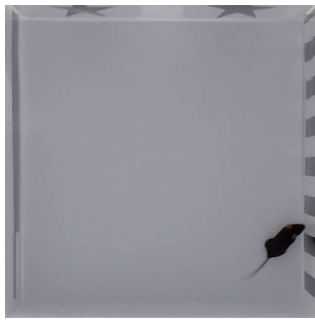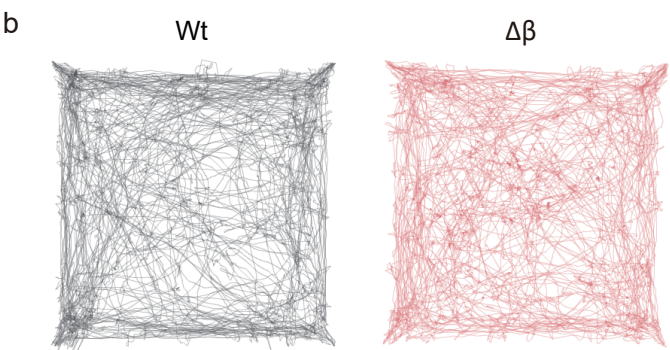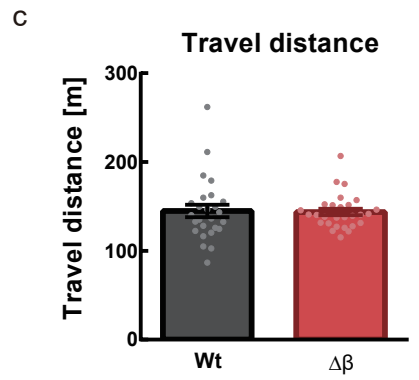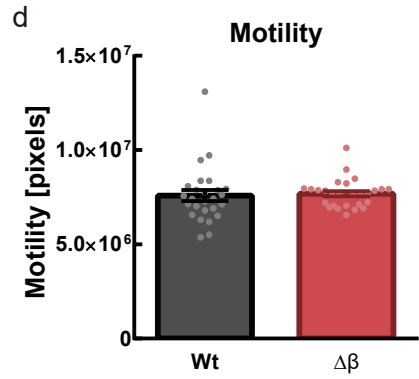

Supplement: Supplementary file 2 — Additional file 2: Figure S2. Behavioral activity was equivalent in open field task. (a) Open field box. (b) Traces of center of mass. Travel distance (c) and motility (d). Panel c: F(25, 27) = 3.434, P = 0.0023; t37.73 = 0.1515, P = 0.880; panel d: F(25, 27) = 4.253, P = 0.0004; t35.54 = 0.2776, P = 0.783; unpaired t-test with Welch’s correction; n = 26 Wt mice, 28 Δβ mice. Data are means ± SEMs. [file 13041_2020_547_MOESM2_ESM.pdf]

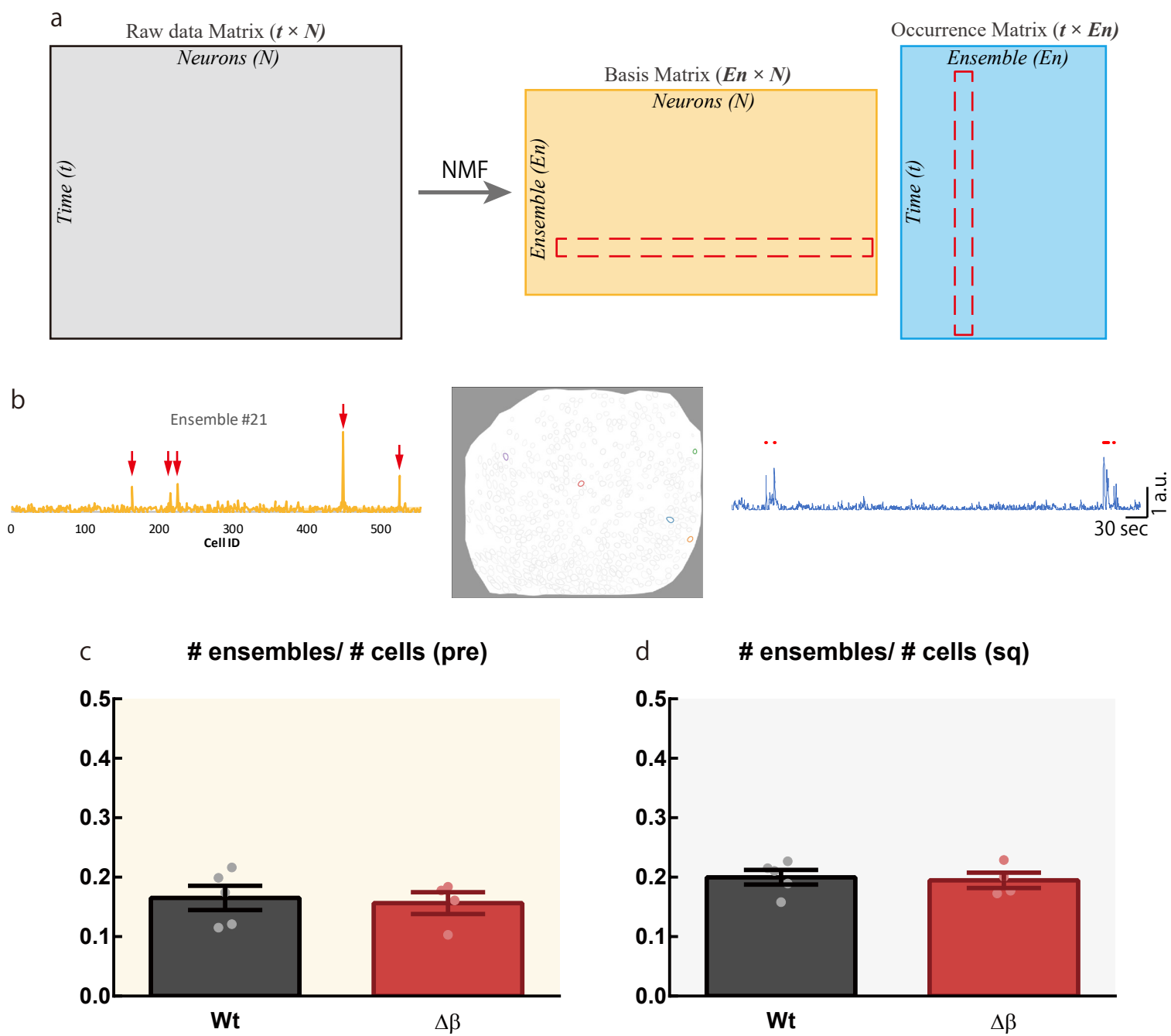

Additional file 4.

Supplement: Supplementary file 4 — Additional file 4: Figure S4. Pcdhβ deficiency does not affect the number of ensembles activated in the home cage and during novel context exploration (related to Fig. 2). (a) Schematic diagram of the non-negative matrix factorization (NMF) analysis. (b) Representative image of an ensemble extracted with NMF. Red arrows (left) indicate the cells contributing to this ensemble. Red dots (right) indicate the times at which the ensemble was activated. (c–d) Normalized number of ensembles extracted in each session (panel c: F(4, 3) = 1.907, P = 0.6229; t7 = 1.648, P = 0.1434; panel d: F(4, 3) = 1.086, P = 0.9856; t7 = 0.2852, P = 0.7837; unpaired t-test; n = 5 Wt mice, 4 Δβ mice). Data are means ± SEMs. [file 13041_2020_547_MOESM4_ESM.pdf]

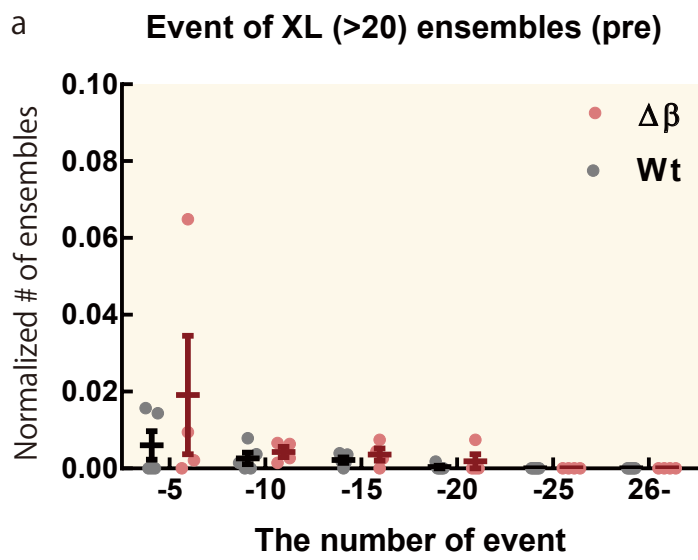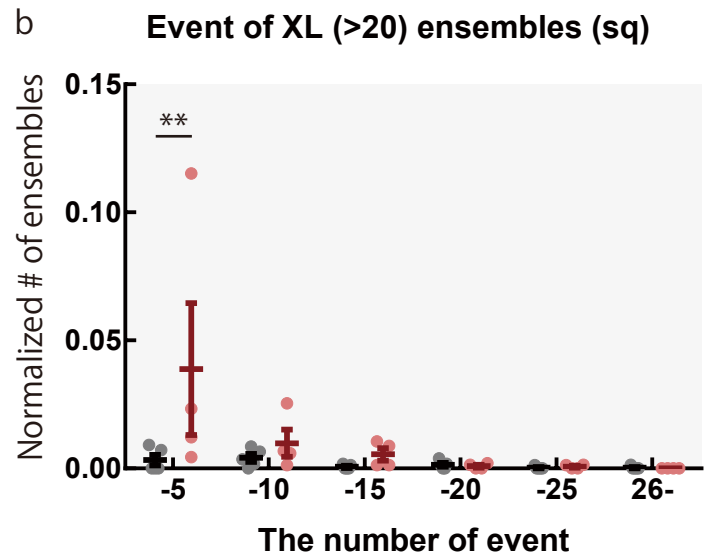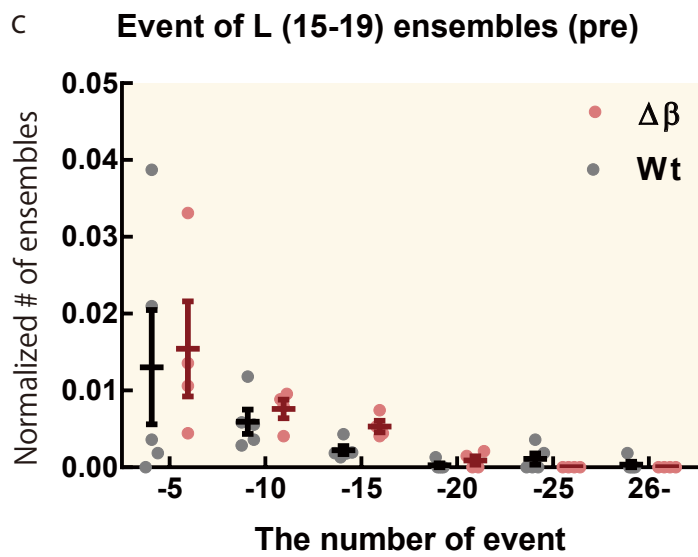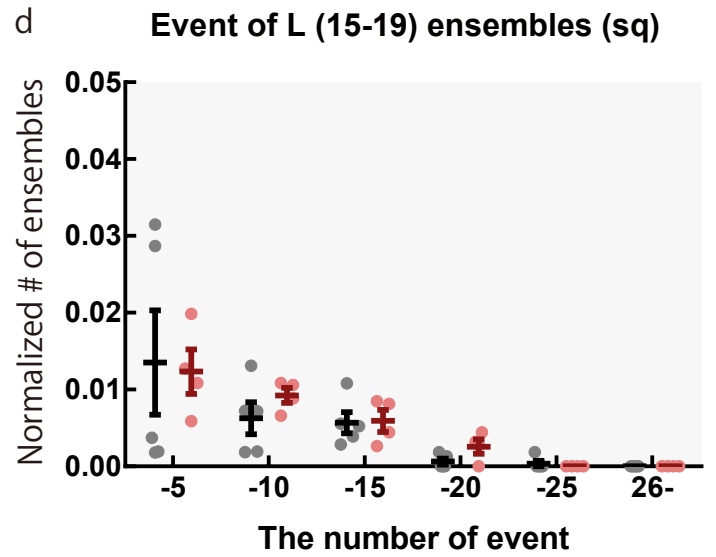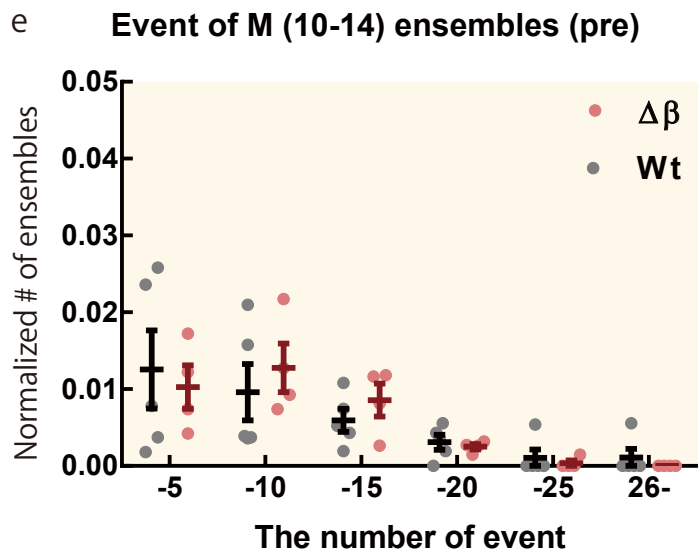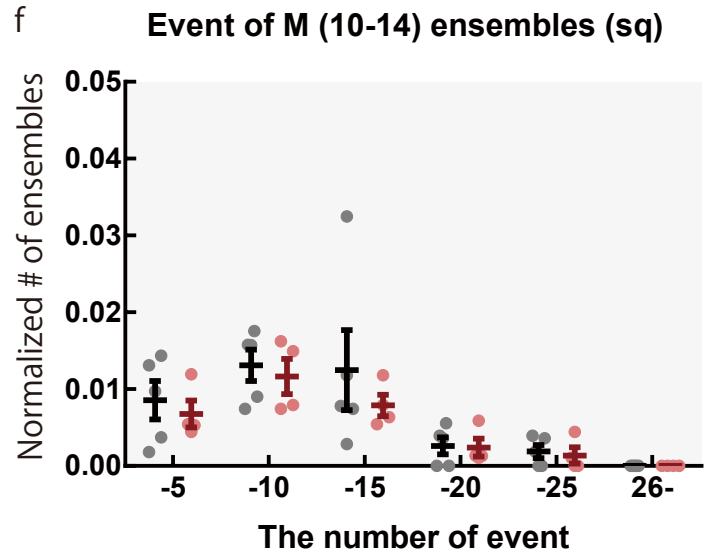

Additional file 5.

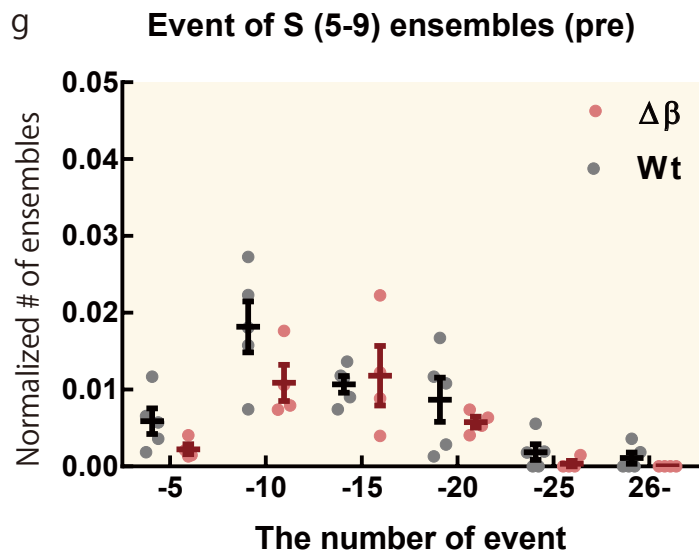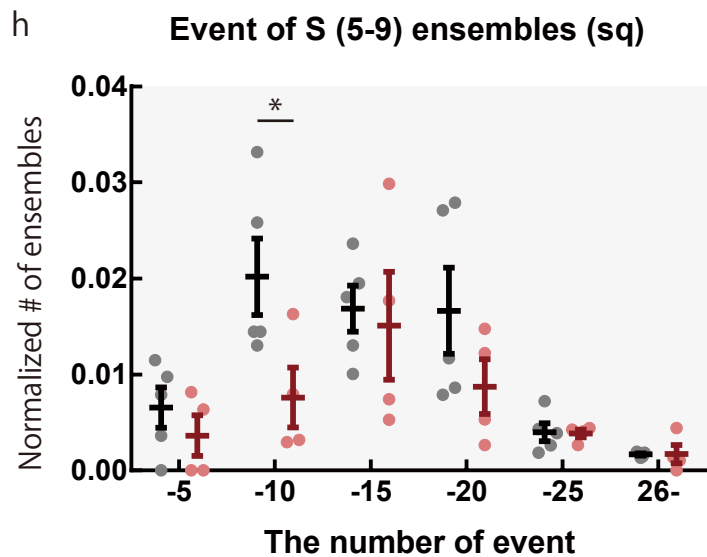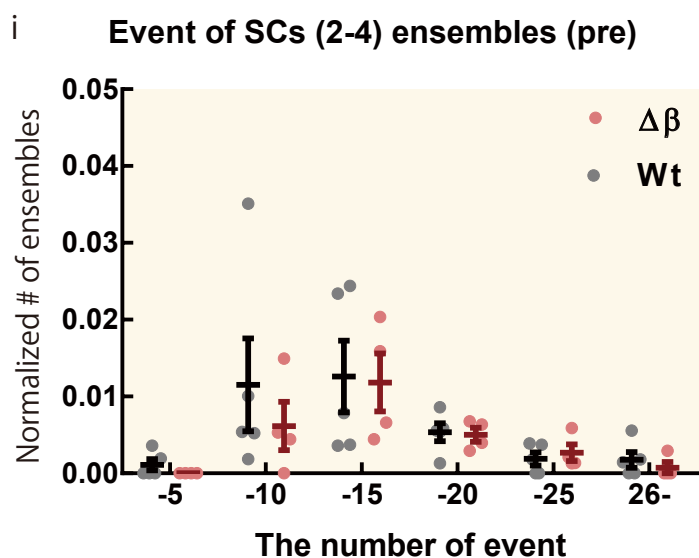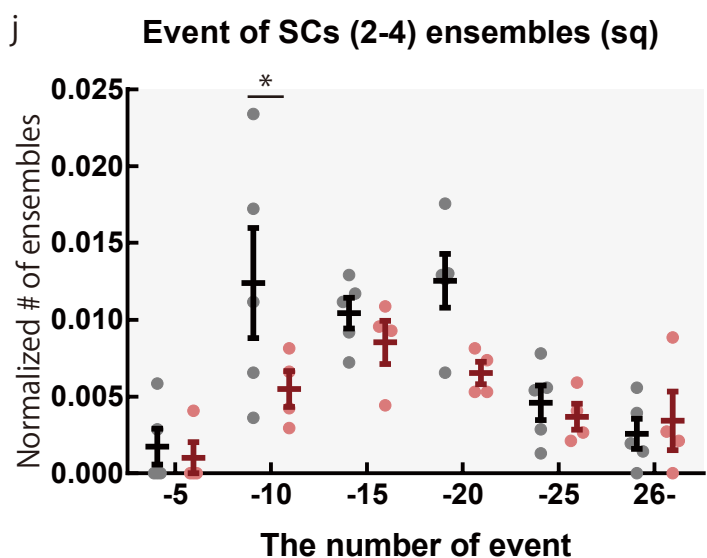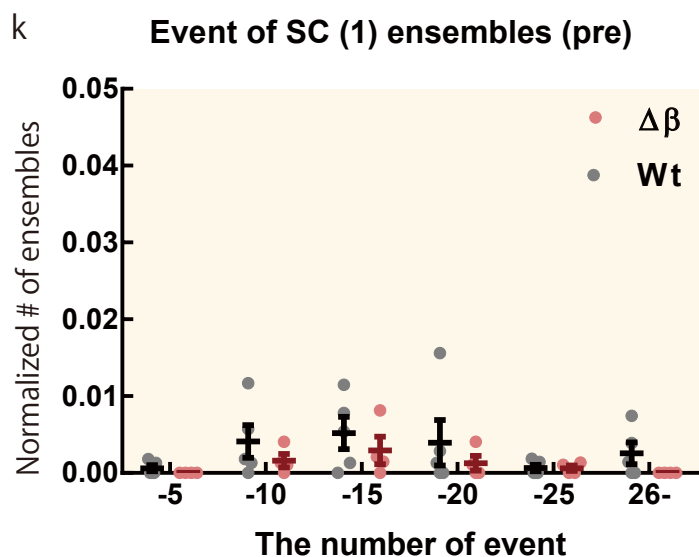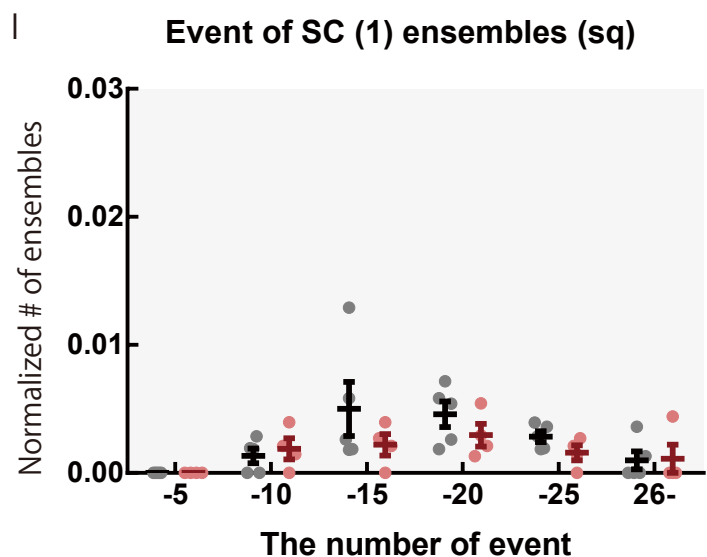

Supplement: Supplementary file 5 — Additional file 5: Figure S5. Large ensembles are infrequently activated. Number of ensemble events during pre and sq sessions in extra-large ensembles (XL; 20 or more cells; a–b), large ensembles (L; 15–19 cells; c–d), medium ensembles (M; 10–14 cells; e–f), and small ensembles (S; 5–9 cells; g–h). Number of ensemble events during pre and sq. sessions in ensembles composed of several cells (SCs; 2–4 cells; i–j) and of single cells (SC; k–l). Statistical values from Bonferroni’s multiple-comparison tests are provided in Additional file 6 (n = 5 Wt mice, 4 Δβ mice). Data are means ± SEMs. *P < 0.05 (adjusted P-value from Bonferroni’s multiple-comparison test). [file 13041_2020_547_MOESM5_ESM.pdf]
