# Supplementary material for: Pcdhβ deficiency affects hippocampal CA1 ensemble activity and contextual fear discrimination
Source: Mol Brain. 2020 Jan 20;13:7. doi: 10.1186/s13041-020-0547-z (PMC6971911; doi:10.1186/s13041-020-0547-z)
Supplement: Supplementary file 3 — Additional file 3: Figure S3. Pcdhβ deficiency does not affect neural activity at the cellular level (related to Figs. 1 and 2, and additional file 1). (a–b) Representative images of raster plots of all recorded cells from Wt (a) and Δβ (b) mice. (c) Cumulative curve of proportion of cells against number of active events during entire recording session (pre 10 min + sq 10 min). Proportions of cells according to the number of Ca2+ events during pre (d) and sq (e) sessions. Statistical values from Bonferroni’s multiple-comparison test are provided in Additional file 6. (f) The ratios of the number of Ca2+ events during the sq session to that in the pre session. Statistical values from Bonferroni’s multiple-comparison test are provided in Additional file 6 (n = 5 Wt mice, 4 Δβ mice). Data are means ± SEMs. No significant differences were observed (Kolmogorov–Smirnov test for panel c, Bonferroni’s multiple-comparison tests for panels d–f). [file 13041_2020_547_MOESM3_ESM.pdf]

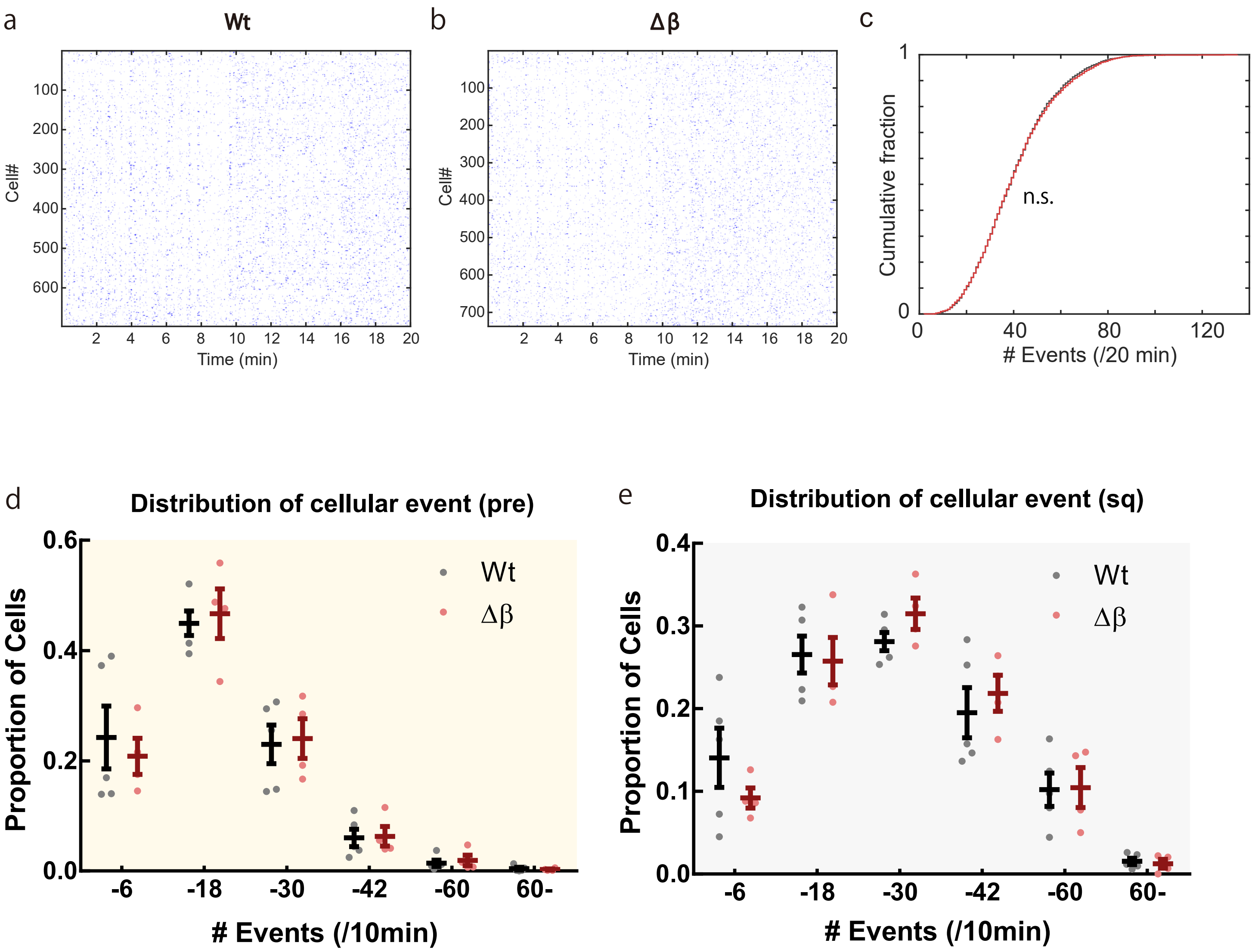

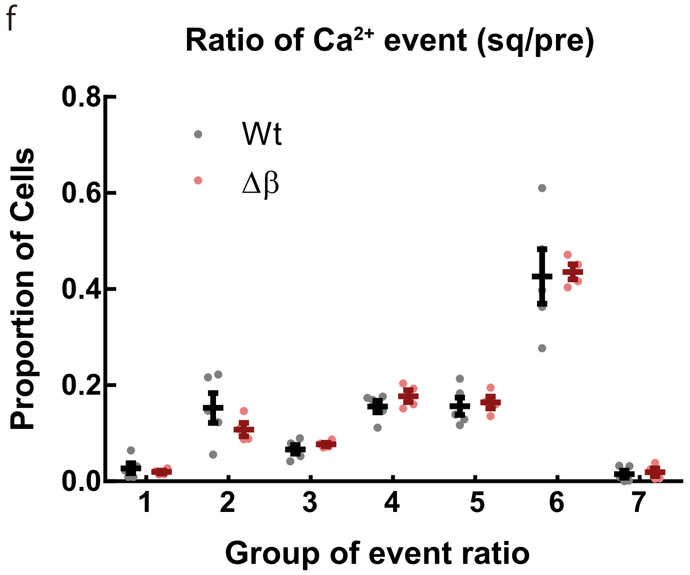

Group	Cellular activity	Ratio
1	Only pre	0
2	pre >> sq	0-.5
3	pre > sq	0.5-.75
4	pre = sq	0.75-1.25
5	pre < sq	1.25-2.0
6	pre << sq	2.0-
7	Only sq	∞

Additional file 3.
